# Angiotensin-Converting Enzyme I/D Polymorphism and Preeclampsia Risk: Evidence of Small-Study Bias

**DOI:** 10.1371/journal.pmed.0030520

**Published:** 2006-12-26

**Authors:** Norma C Serrano, Luis A Díaz, Maria C Páez, Clara M Mesa, Rodrigo Cifuentes, Alvaro Monterrosa, Adriana González, Liam Smeeth, Aroon D Hingorani, Juan P Casas

**Affiliations:** 1 Universidad Autónoma de Bucaramanga, Bucaramanga, Colombia; 2 Instituto de Ciencias de la Salud, Medellín, Colombia; 3 Universidad del Valle, Cali, Colombia; 4 Universidad de Cartagena, Cartagena, Colombia; 5 Universidad Industrial de Santander, Bucaramanga, Colombia; 6 Department of Epidemiology and Population Health, London School of Hygiene and Tropical Medicine, United Kingdom; 7 Centre for Clinical Pharmacology, Department of Medicine, at British Heart Foundation laboratories at University College London, London, United Kingdom; University of Leicester, United Kingdom

## Abstract

**Background:**

Inappropriate activation of the renin–angiotensin system may play a part in the development of preeclampsia. An insertion/deletion polymorphism within the angiotensin-I converting enzyme gene *(ACE-I/D)* has shown to be reliably associated with differences in angiotensin-converting enzyme (ACE) activity. However, previous studies of the *ACE-I/D* variant and preeclampsia have been individually underpowered to detect plausible genotypic risks.

**Methods and Findings:**

A prospective case-control study was conducted in 1,711 unrelated young pregnant women (665 preeclamptic and 1,046 healthy pregnant controls) recruited from five Colombian cities. Maternal blood was obtained to genotype for the *ACE-I/D* polymorphism. Crude and adjusted odds ratio (OR) and 95% confidence interval (CI) using logistic regression models were obtained to evaluate the strength of the association between *ACE-I/D* variant and preeclampsia risk. A meta-analysis was then undertaken of all published studies to February 2006 evaluating the *ACE-I/D* variant in preeclampsia. An additive model (per-*D*-allele) revealed a null association between the *ACE-I/D* variant and preeclampsia risk (crude OR = 0.95 [95% CI, 0.81–1.10]) in the new case-control study. Similar results were obtained after adjusting for confounders (adjusted per-allele OR = 0.90 [95% CI, 0.77–1.06]) and using other genetic models of inheritance. A meta-analysis (2,596 cases and 3,828 controls from 22 studies) showed a per-allele OR of 1.26 (95% CI, 1.07–1.49). An analysis stratified by study size showed an attenuated OR toward the null as study size increased.

**Conclusions:**

It is highly likely that the observed small nominal increase in risk of preeclampsia associated with the *ACE D*-allele is due to small-study bias, similar to that observed in cardiovascular disease. Reliable assessment of the origins of preeclampsia using a genetic approach may require the establishment of a collaborating consortium to generate a dataset of adequate size.

## Abstract 2

The observed small increase in risk of preeclampsia associated with the*ACE D*-allele is likely to be due to small-study bias, a similar result to that observed in cardiovascular disease.

## Editors' Summary

**Background.:**

Preeclampsia is a common condition affecting pregnant women worldwide; it is defined as the presence of increased blood pressure, together with protein in the urine. Although in many women preeclampsia may never result in symptoms, other women may experience headaches, problems with their vision, swollen ankles and feet, and other problems. Sometimes, preeclampsia progresses to eclampsia, in which potentially life-threatening seizures result. The causes of preeclampsia are not well understood, but several factors are known to contribute to the risk. These factors include diabetes, high blood pressure prior to pregnancy, obesity, and first pregnancy. There is also the possibility that preeclampsia has, at least in part, a genetic basis; the condition is more likely among women whose relatives have also had it. However, no definite genetic cause has yet been confirmed.

**Why Was This Study Done?:**

A common variant in one particular gene, *ACE,* which codes for the angiotensin-1 converting enzyme, has been linked with preeclampsia in a number of different studies. The protein encoded by *ACE* is involved in controlling blood pressure and the balance of fluid and salts in the blood. However, many of the studies supposedly linking *ACE* and preeclampsia were done on very few participants. Small studies are more likely to generate “false positive” findings. Therefore, a group of investigators from Colombia and the UK wanted to find out whether they could reproduce the supposed link between the *ACE* gene variant and preeclampsia in a large study, and also to see whether the previous studies could have been “false positives.”

**What Did the Researchers Do and Find?:**

These investigators carried out a case-control study. This means that women with preeclampsia (“cases”) were recruited, and compared with women similar in all other respects but who did not have preeclampsia (“controls”). In total 1,711 pregnant women from five Colombian cities were studied, of whom 665 had preeclampsia and 1,046 did not. Blood was taken from each participant and used for DNA sequencing of the *ACE* gene. The investigators then did a statistical comparison to see whether there was any association between preeclampsia and possession of a particular variant of the *ACE* gene. The results showed that there was no such association. Then, the investigators did a literature search to find all previous studies that had examined a possible link between variants of the *ACE* gene and preeclampsia. They found 22 studies reporting data obtained from 6,424 women (these figures include the results from the investigators' own case-control study described here). The data from all of these studies were then put together into a combined analysis. This combined analysis did suggest a small increase in the risk of preeclampsia in women with one particular variant in the *ACE* gene. However, this result was more likely in studies with small numbers of participants. Furthermore, the earliest studies done were most likely to show an effect, with the supposed link disappearing as more and more data were collected.

**What Do These Findings Mean?:**

The findings presented here suggest that “small study bias” may explain the discrepancy between the results of the case-control study and the combined analysis. That is, studies involving few participants are less reliable and more likely to produce false-positive results. Therefore, it is possible that the proposed link between *ACE* gene variants and preeclampsia is a spurious one. The investigators propose that in future, collaborative research networks will be needed to carry out rigorous research on the genetics of preeclampsia. Such initiatives will help to overcome the problem of bias that can arise from small studies.

**Additional Information.:**

Please access these Web sites via the online version of this summary at http://dx.doi.org/10.1371/journal.pmed.0030520.

- Information for patients from NHS Direct (UK National Health Service) about preeclampsia
- Medical encyclopedia entry on preeclampsia from MedLine Plus, supplied by the US National Library of Medicine
- Information from the World Health Organization and Pan American Health Organization on maternal health in the Americas

## Introduction

Preeclampsia is a maternal disease of pregnancy associated with increased blood pressure and proteinuria after 20 weeks of gestation. It is a major cause of maternal and neonatal mortality and morbidity worldwide and has particularly high incidence in Latin American and Caribbean countries, in which hypertensive disorders during pregnancy account for 25.7% of maternal deaths [1,2]. Preeclampsia is thought to be the result of the interplay between important genetic components and environmental influences; however, the factors and the mechanisms that lead to preeclampsia remain elusive [3]. As a result, there is a lack of effective preventive interventions [4].

With the exception of smoking, established risk factors for cardiovascular disease, including high blood pressure, diabetes, and obesity, are also risk factors for preeclampsia [5]. In addition, women who suffer from preeclampsia have an increased risk of later cardiovascular disease, which clearly suggests a shared aetiology [6].

Inappropriate activation of the renin–angiotensin system may play a part in the development of many cardiovascular disorders, including preeclampsia [7,8]. A common insertion/deletion polymorphism within the angiotensin-I converting enzyme gene *(ACE-I/D)* has been reliably associated with substantial differences in the plasma and tissue angiotensin-converting enzyme (ACE) activity in a codominant (additive) fashion not only in persons of European descent, but also in other populations such as Hispanics [9–11]. Individuals carrying the *D* allele have higher ACE activity, which has been proposed as an intermediate phenotype of potential relevance for the development of high blood pressure and subclinical atheroma (i.e., higher intima-media thickness of the carotid artery) [10,12]. Despite the biological plausibility and the consistency of the effect of the *ACE-I/D* polymorphism on ACE activity, associations of the *ACE-I/D* polymorphism and coronary heart disease, coronary artery restenosis, stroke, and renal disease have been inconsistent [13–16]. Moreover, systematic reviews and meta-analyses have indicated the presence of small-study bias in the published literature [13–16].

Several studies, also usually small in size, have reported that women carrying the *D* allele of the *ACE-I/D* polymorphism have higher ACE activity and higher measures of uterine artery resistance, which is a marker for development of intrauterine growth retardation and preeclampsia [8,17]. These observations led to the proposal that the *ACE-I/D* polymorphism may be a good candidate in the search for a cause of preeclampsia. However, to date, studies evaluating the role of *ACE-I/D* polymorphism in preeclampsia have been individually underpowered to detect plausible genetic effect sizes, being much smaller than more recent studies in cardiovascular disease. We hypothesized that the published literature on the *ACE-I/D* polymorphism in preeclampsia might be similarly affected by small-study bias. To test this hypothesis, we conducted a new large genetic association study on the *ACE-I/D* polymorphism and preeclampsia in a geographical region with a high incidence of preeclampsia. We then set this study within the context of a systematic review and a meta-analysis of all studies conducted to date.

## Methods

### Case-Control Study Participants

A prospective case-control study was conducted in 1,711 unrelated young pregnant women (665 preeclamptic participants and 1,046 healthy pregnant controls) recruited from five Colombian cities at the time of delivery between January 2000 and December 2005. A verbal interview with a structured questionnaire was conducted by trained personnel at the time of the delivery to ascertain maternal age, gestational age, parity, smoking status during pregnancy, family history of preeclampsia, ethnic background, and socioeconomic position. On two separate occasions, the mean of two readings of blood pressure was obtained (four measurements in total) at the time of delivery. Blood pressure was measured in the right arm after a five-minute period of rest in a seated position using mercury sphygmomanometers or electronic devices calibrated against a mercury standard.

A case was defined as a primigravid woman younger than 26 years old with blood pressure 140/90 mm Hg or above, and proteinuria 0.3 grams or above in 24 hours, or a reading 2+ or above on a dipstick in a random urine determination with no evidence of urinary tract infection after 20 weeks of gestation [18]. At least one control was recruited after each case within a window of 24 hours from the same hospital that provided the cases. A control was defined as a primigravid woman younger than 26 years old without preeclampsia and in labour after 37 weeks of pregnancy. To improve the homogeneity of the phenotype under evaluation, women with a prior history of autoimmune, metabolic (including diabetes or gestational diabetes), renal, or cardiac (including chronic hypertension) diseases were excluded from the study. All participants signed the informed consent document approved by the Ethics Committee from the Universidad Autónoma de Bucaramanga, Colombia.

#### DNA extraction and genotyping.

Blood was drawn from the antecubital vein into EDTA and samples stored at −50 °C for DNA extraction, using the QIAamp DNA blood mini-kit (QIAGEN, Hilden, Germany). The *ACE-I/D* polymorphism in intron 16 was detected according to the method described by Rigat, et al. [9]. PCR is known to have a tendency to preferentially amplify the short deletion *(D)* allele in contrast to the larger insertion *(I)* allele in a competitive amplification reaction when both alleles are present, as occurs in individuals with the *ID* genotype. This leads to mistyping of *ID* individuals as *DD* in approximately 4%–5% of the samples. To avoid any mistyping of *ID* as *DD,* a second PCR amplification using insertion-specific primers was conducted for all participants who were homozygous for the *D* allele [19]. For a detailed description of the genotyping methods, see Protocol S1. All available DNA samples were genotyped and included in the present report, with no exclusions. For quality control, a random sample (*n* = 156) was subjected to a second PCR and genotyping to minimize any possible misclassification. The Cohen's kappa value among the samples regenotyped was equal to 0.95 (95% CI [confidence interval], 0.94–0.96), and the error rate was 2.56% (95% CI, 0.70–6.43). Genotyping was conducted blinded to the clinical status of the participants.

#### Statistical analysis.

To evaluate the presence of differences between groups, unpaired Student's t-, χ^2^, or Mann-Whitney tests were used as appropriate. A test for departure from Hardy-Weinberg equilibrium was performed by *χ* ^2^ analysis. The principal a priori hypothesis was that the association between the *ACE-I/D* polymorphism and preeclampsia follows an additive model according to the number of *D* alleles. The additive “per-allele” model (in a log-scale) was based on the effect of the *ACE-I/D* variant on its intermediate phenotype (ACE activity), established in large studies and overviews as being a reliable association [10]. However, recessive and dominant models and multiple pairwise comparisons (*ID* versus *II* and *DD* versus *II*) were also evaluated for completeness as secondary outcomes.

Multivariate analysis using logistic regression methods was also conducted to control for potential confounders (maternal age, ethnic background, place of birth of the women, recruitment centre, socioeconomic position, urinary or vaginal infections during pregnancy, and smoking status during pregnancy).

To explore the prior hypothesis that the genotypic odds ratio (OR) is greater in women with an enriched phenotype, an analysis using an additive model was repeated for women stratified according to the presence of family history of preeclampsia (positive versus negative family history in mother or sisters), and disease severity with severe preeclampsia being defined as blood pressure above 160/110 mm Hg or proteinuria of 5 grams or more in 24 hours, eclampsia, or the HELLP syndrome. For these analyses 99% CIs were used to make some allowance for their exploratory nature. All statistical analyses were conducted using Stata, Version 9 (Stata Corporation, College Station, Texas, United States).

### Meta-Analysis

#### Search strategy and selection criteria.

Electronic databases (MEDLINE, EMBASE, LILACS, KoreaMed, and Google Scholar) were searched up to February 2006 for all genetic association studies evaluating the *ACE-I/D* polymorphism and preeclampsia in humans in all languages. The search strategy contained both medical subject heading terms and text words as follows: “angiotensin-converting enzyme” or “ACE” or “peptidyl-dipeptidase A,” in combination with “pre-eclampsia” or “preeclampsia” or “pregnant hypertensive disorders” or “pregnancy hypertension,” and combined with “genetic” or “polymorphism(s)” or “mutation” or “genotype” or “gene(s).” No limits were used in the search strategy. We searched for any additional studies in the references of all identified publications, including previous relevant meta-analyses, and used the MEDLINE option “related articles” for all the relevant papers.

For inclusion, studies had to involve unrelated women and examine the association between the *ACE-I/D* polymorphism and preeclampsia. Studies published as full-length articles or letters in peer-reviewed journals in any language were included, as well as abstracts taken from reference lists of identified publications. Authors were contacted (on at least three occasions) to obtain information on the genotype frequency by case-control status and by disease severity (severe and nonsevere preeclampsia), the use of blinding of genotyping staff to clinical status, the definition of outcomes, and, in a few cases, to clarify possible overlapping of study results. A positive reply was obtained in 15 out of 21 study authors contacted.

#### Data extraction.

The following information was extracted (entered into databases by two of the authors, JPC and MCP) from each study and disagreements resolved by consensus: year of publication, total cases, total controls, number of individuals by each genotype, study design, source of controls, matching variable, thresholds used to define preeclampsia, country of origin, ethnicity, *χ* ^2^ goodness of fit for Hardy-Weinberg equilibrium and its *p*-value, use of blinding, mean age of participants, frequency of nulliparous women, and main exclusion criteria. In the few instances in which genotype frequencies provided by the investigators in tabular data differed slightly from published figures, the tabular data were used. Women with gestational hypertension were excluded from the present meta-analysis in order to improve the homogeneity of phenotype between studies.

#### Statistical analysis.

Data were analysed using Stata 9. The genetic model to be considered as the priori hypothesis was an additive model, following the considerations previously described for the case-control study. Secondary analyses involved ORs for other genetic models of inheritance such as recessive, dominant, and pairwise comparisons of the different genotypes. For all the models used, the *D* allele was considered the one at risk. For the additive or per-allele model, the OR was compared between cases and controls by assigning scores for different genotype groups and calculating ORs by logistic regression. We calculated the random effect summary OR and CIs for each polymorphism. To make some allowance for multiple comparisons 99% CIs were used for individual studies, and 95% CIs were reserved for the combined estimates. The inverse variance-weighted method was used to calculate the summary OR [20]. Heterogeneity was assessed by the DerSimonian and Laird *Q* test and *I* ^2^ was used as a measure to describe the percentage of variability in point estimates that was due to heterogeneity rather than sampling error [20]. Sources of between-study heterogeneity were explored using random effect metaregression models with restricted maximum likelihood estimation. The prespecified characteristics for assessment of sources of inter-study heterogeneity were: Study size (number of cases: <100, 100–200, and ≥200); blinding of genotyping staff (blinded, unblinded, or unknown); disease severity (severe versus nonsevere preeclampsia); ethnicity of women evaluated (of European descent, Asian, Hispanic, Afro-Caribbean, and others); preeclampsia definition (adequate versus unclear); and publication language (English– versus non-English–language journals). Funnel plots of the effect estimate against the sample size, Egger regression asymmetry test, and a weighted (by the inverse of the variance of the estimate) linear fixed regression of the log OR against the inverse of the sample size (linear-regression model) were used to evaluate small-study effects [20,21]. To evaluate stability over time of the effect estimate, cumulative meta-analysis using random effect models was conducted [20].

## Results

### Case-Control Study

Clinical and demographic data of the cases and controls are shown in Table 1. There were no significant differences in maternal age, ethnic background, and socioeconomic position between cases and controls (Table 1). Expected differences in maternal blood pressure, parity, and newborn weight and condition were recorded.

**Table 1:**
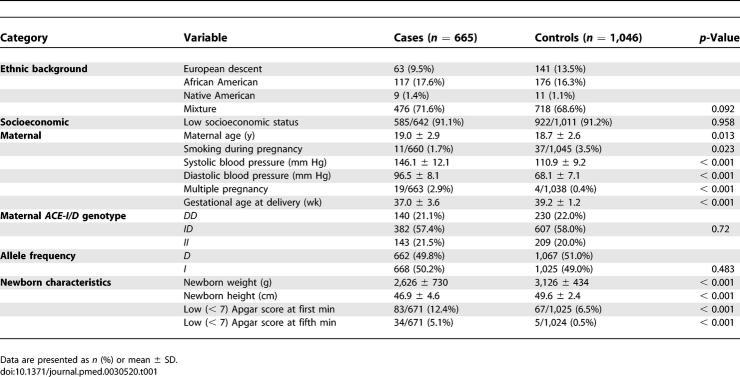
Maternal and Neonatal Characteristics of the Sample Studied

**Figure 1:**
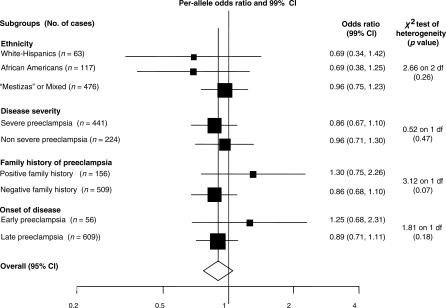
Risk Association of *ACE-I/D* Variant with Preeclampsia in Selected Subgroups within the Current Study

The distribution of the *ACE-I/D* genotypes and allelic frequencies were not significantly different according to the case-control status (Table 1). Genotype frequencies according to the ethnic group and by recruitment centre are reported in “Appendix Table I” in Protocol S1. An additive model (per-*D*-allele) revealed a null association between the *ACE-I/D* variant and preeclampsia risk (crude OR = 0.95 [95% CI, 0.81–1.10]). Adjusting for maternal age, ethnicity, recruitment centre, and place of birth, aimed to minimize the effect of possible population admixture, did not change the estimate of the effect (model-1 OR = 0.92 [95% CI, 0.78–1.07]). A similar result was obtained after further adjustment for additional potential confounders such as socioeconomic position, presence of urinary or vaginal infections during pregnancy, and smoking status during pregnancy (model-2 OR = 0.90 [95% CI, 0.77–1.06]). ORs for other genetic models of inheritance also yielded a null association (Table 2). Pre-specified exploratory subgroup analyses indicated that with the exception of family history of preeclampsia (positive-history OR = 1.30 [99% CI, 0.75–2.26] versus negative-history OR = 0.86 [99% CI, 0.68–1.10]; *p*-value for heterogeneity equal to 0.07), no substantial heterogeneity of the genetic effect size was observed for any of the subgroups (Figure 1). Additionally, stratified analysis by recruitment centre according to the conformity with Hardy-Weinberg equilibrium yielded similar, null results (see “Appendix Table II” in Protocol S1).

**Table 2:**
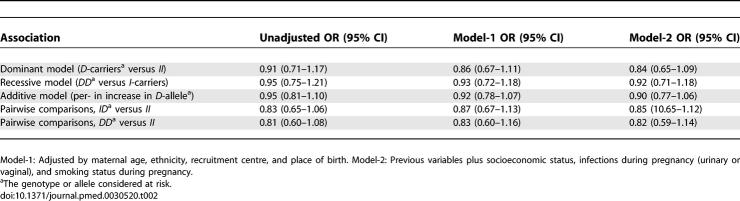
Estimate of the Effect of the *ACE-I/D* Polymorphism on Preeclampsia Risk Modeled with Logistic Regression

### Meta-Analysis

A total of 30 genetic association studies, including the present study, evaluating the *ACE-I/D* gene variant and preeclampsia risk were identified [17,22–49]. We excluded eight out of 30 studies for one or more of the following reasons: two in which duplication or partial overlapping of reported data were considered likely [43,44]; three in which the outcome evaluated was solely gestational hypertension [45–47]; one in which relevant data were not reported and could not be obtained from study authors [48]; one that only recruited women with previous preeclampsia [17]; and one in which the sampling frame was based on the *ACE-I/D* genotype [49]. A total of 22 genetic association studies including 2,596 cases and 3,828 controls were included in the present meta-analysis (Tables 3 and 4) [22–42]. Out of the 22 studies, nine were conducted with Asian participants, eight with participants of European descent, two with African Americans, two with more than one ethnic group, and one with South Asians.

**Table 3:**
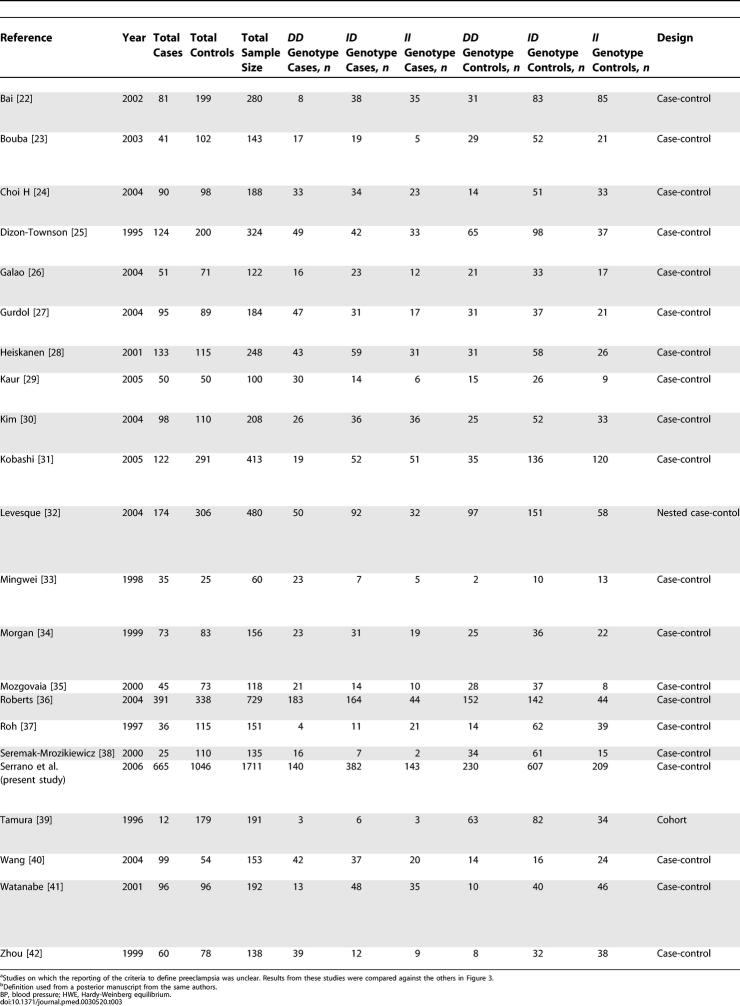
Characteristics of Published Studies of the Association between the *ACE-I/D* Polymorphism and Preeclampsia Included in the Meta-Analysis

**Table 3:**
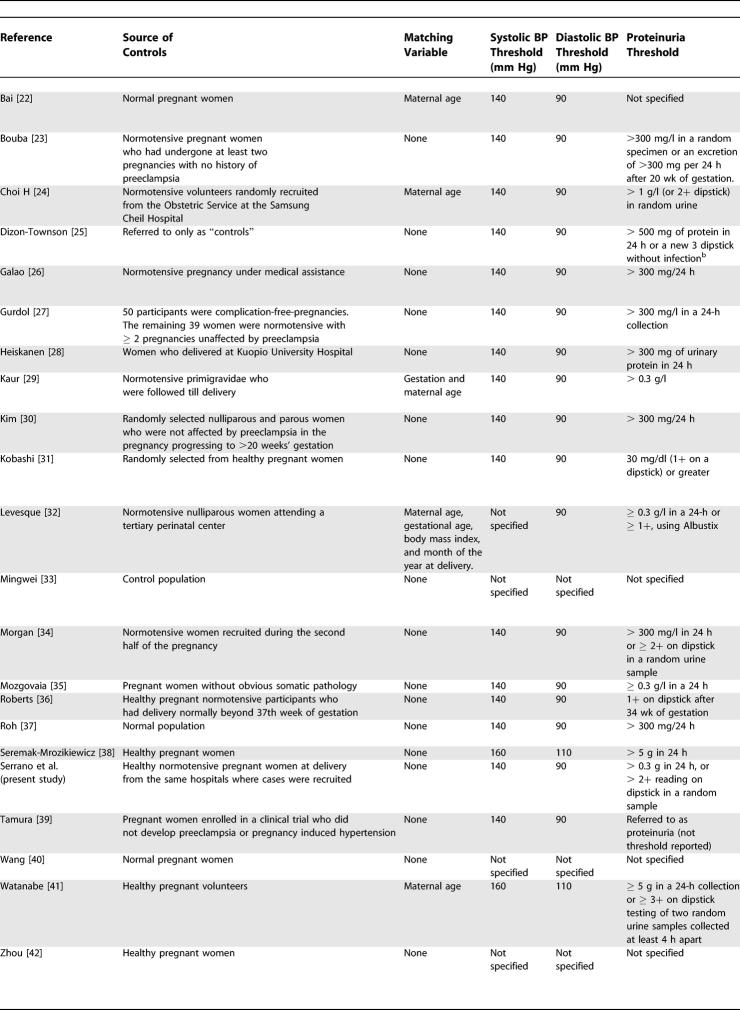
Extended.

**Table 3:**
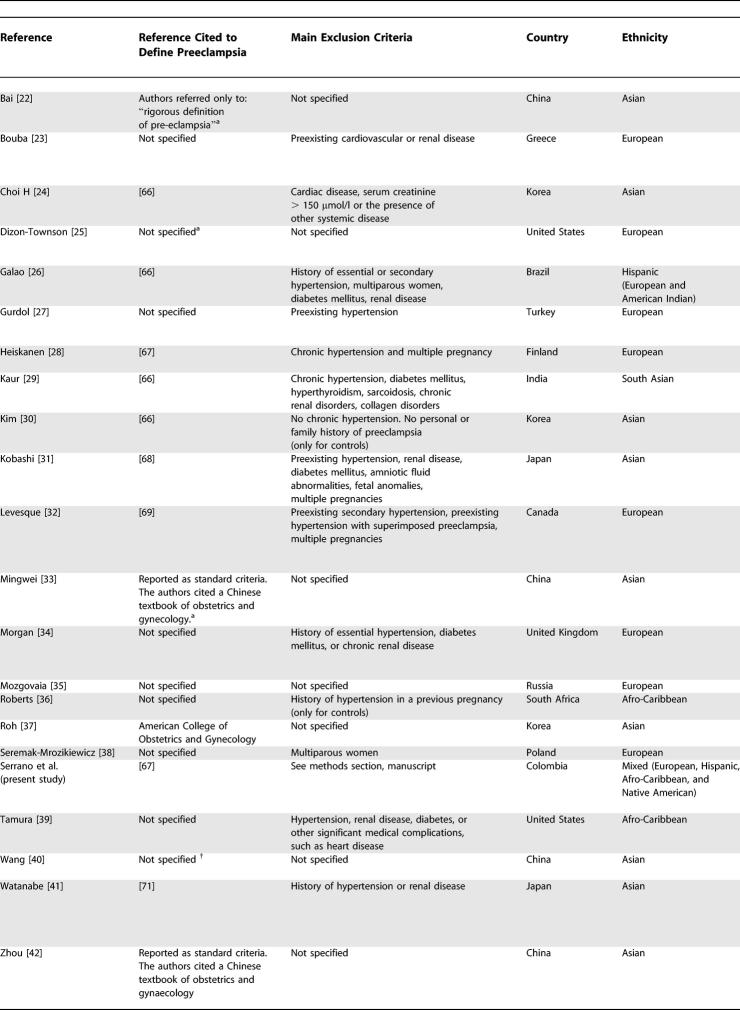
Extended.

**Table 4:**
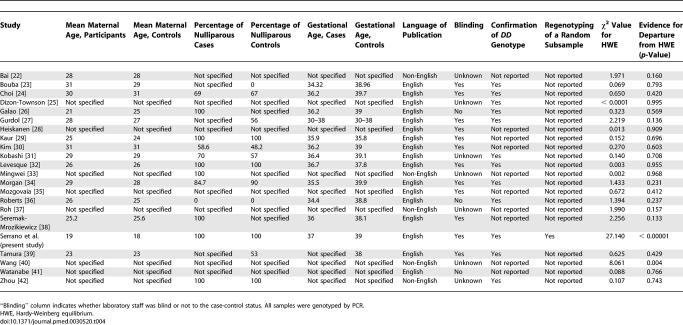
Characteristics of Published Studies of the Association between the *ACE-I/D* Polymorphism and Preeclampsia Included in the Meta-Analysis

**Figure 2:**
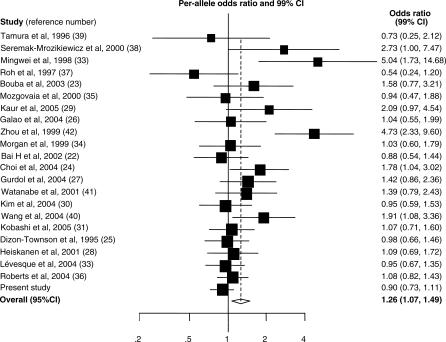
Meta-Analysis of Studies of *ACE-I/D* Polymorphism and Risk of Preeclampsia

**Figure 3:**
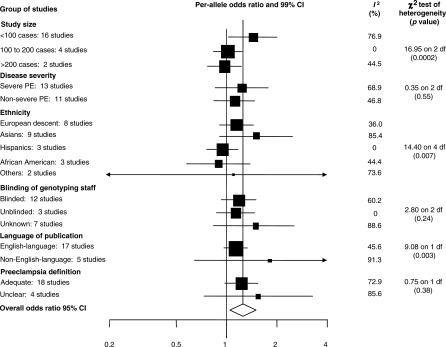
Studies of *ACE-I/D* Polymorphism and Risk of Preeclampsia Grouped by Study Characteristics

The OR under an additive model for preeclampsia was 1.26 (95% CI, 1.07–1.49; *p* = 0.006) (Figure 2). However, there was evidence of substantial between-study heterogeneity (*I* ^2^ = 75.1%, *χ_21_* ^2^ = 84.23, *p*_Het_ < 0.0001). Study characteristics such as blinding of genotyping staff, publication language, preeclampsia definition, and disease severity explained little of the heterogeneity (Figure 3). A stratified analysis by study size, evaluated as the number of cases in each study (<100, 100–200, and ≥200), showed a diminished effect as the study size increased (*χ_2_* ^2^ = 16.95, *p*_Het_ = 0.0002) (Figure 3). Analogous results were obtained when different cutoff points (< 100, 100–500, and ≥ 500) for the number of cases were used (*χ_2_* ^2^ = 18.51, *p*_Het_ = 0.00009). Similarly, stratifying by ethnicity indicated that studies conducted in Asian populations tended to have a larger ORs (*χ_5_* ^2^ = 14.4, *p*_Het_ = 0.007). These findings might be explained by the fact that eight out of the nine studies conducted in Asian populations had fewer than 100 cases in each study. The funnel plot including all studies was asymmetric, and the Egger's test (*p* = 0.06) and the linear regression model (*p* = 0.003) suggested the presence of an excess of small studies with more positive results, predominately of studies published in non-English–language journals (Figure S1; see Protocol S1 for details). A cumulative synthesis of ACE-I/D variants and preeclampsia revealed substantial instability of the genetic effect over time, with studies published between 1996 and 1997 demonstrating the most protective effect, immediately followed by studies published in 1998 and 1999 indicating the most significant results in the opposite direction. Then, the effect size is seen to attenuate gradually over time toward a null association with the accumulation of more data (Figure S2; see Protocol S1 for details). Genotypic ORs under other genetic models of inheritance are outlined in Table 5.

**Table 5:**
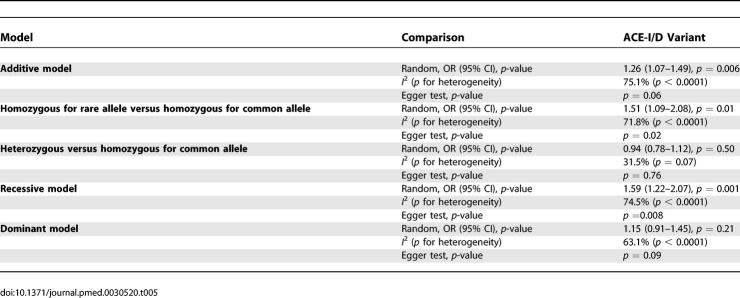
Genotypic ORs for Preeclampsia and the *ACE-I/D* Variant.

## Discussion

The current meta-analysis, which includes new data from the largest case-control study to date, represents the most comprehensive evaluation of the *ACE-I/D* variant in preeclampsia. Although a pooled per-allele OR suggested evidence of an increase in the risk of preeclampsia of 1.26 (95% CI, 1.07–1.49), the robustness of this summary estimate is uncertain. First, our study found a null association of the *ACE-I/D* variant with preeclampsia. Moreover, there was no substantial evidence of a positive effect in any of the subgroups in the prespecified analyses. Second, the meta-analysis revealed diminishing summary risk estimate as study size increased (Figure 3), regardless of the arbitrary cutoff points used to define the categories. This result is concordant with the results of several statistical tests used to evaluate the presence of small-study bias (Egger's test *p* = 0.06 and linear-regression model test *p* = 0.003). Furthermore, since only the published literature was included, it is possible that including unpublished studies (which more often provide evidence of negative or null effects) would have provided additional evidence of small-study bias. Taken together, these findings point to small-study bias as a potential explanation for the results observed in the meta-analysis. Discrepant findings from large and small studies are not new in the field of genetics of complex disorders [50]. When present, discrepancy of genetic effects sizes may be due to multiple causes such as genuine heterogeneity, data manipulation and fabrication, study quality, or publication bias. A form of publication bias relevant to the current report is within-study reporting bias. Because of the facility of measuring multiple genetic markers in a study, significant positive and negative associations (sometimes arising from multiple testing) are more likely to be published early rather than late. Results from the cumulative meta-analysis support this as one possible explanation, which has been referred to as the Proteus phenomenon [51]. Further evidence in support of the presence of within-study reporting bias is the fact that studies published in languages other than English, and in the Asian ethnic group, tend to have larger effects, findings consistent with other recent results [52].

Publication bias is increasingly being recognized as one of the main threats to the reliability of conclusions drawn from association studies with common disease outcomes. In the setting of cardiovascular and neurological diseases, several positive gene–disease associations, usually based on meta-analysis of small studies, have been subsequently refuted by large genetic studies [13,53]. As a result, several initiatives are now underway to help overcome problems of reporting and publication bias and to help to achieve datasets of appropriate size to detect plausible genetic effects for common disorders, which are likely to require several thousands of cases of the disease (The Wellcome Trust Case Control Consortium, http://www.wtccc.org.uk) [54]. Genetic studies in preeclampsia continue to be somewhat small in size [55,56] and are usually underpowered to detect realistic genotypic relative risks (ORs between 1.15 and 1.4) [14,57]. However, considering the low incidence of preeclampsia (2%–3% in developed countries), it is highly unlikely that a single centre will be able to amass the large number of cases required, and the development of networks of interested investigators may be essential [54]. Therefore, international collaborations, particularly among those countries with a high incidence of preeclampsia, may make recruitment more efficient and help to include participants with different cultural and genetic backgrounds, which can provide further insight into the aetiology of the disease both genetic and/or environmental.

Despite these obstacles, the investment in adequate resources to study the genetics of preeclampsia is an important priority. Observational studies and the randomised trials of interventions that have followed have been unsuccessful thus far in identifying causal pathways in preeclampsia amenable to preventive therapies, a clear example of which are the recently failed clinical trials using either antioxidant vitamins or calcium supplements [58–61]. A genetic approach that is less prone to confounding and reverse causation than nongenetic observational studies, may be more likely to identify causal pathways and may help to prioritise therapeutic targets that require evaluation in large and expensive randomised clinical trials [62,63]. The challenge is in how to make better use of the genetic approach in complex diseases such as preeclampsia, in particular to overcome random errors in risk estimates from small studies as well as publication bias. A suggested approach is to establish a collaborating consortium of investigators from existing studies in genetics of preeclampsia to reduce the multiple existing problems such as: (1) inadequate selection of candidate gene variants to be evaluated, (2) biased analyses, and selective reporting of positive results; (3) to promote access to unpublished data; (4) to overcome inadequate outcome definitions; and (5) to provide guidance for developing new large studies [54]. Until such measures are established, it will be important for both authors and journal editors to embrace the publication of both positive and negative results from “well-designed case-control” studies to diminish the problem of publication bias [64]. This approach has recently become a reality for clinical trials [65], and it might help in reducing the temptation of researchers to explore multiple hypotheses in subgroup analyses to obtain one finding of nominal statistical significance that might help acceptance of the paper.

Investigating the aetiology of preeclampsia, one of the main causes of maternal and neonatal mortality and morbidity worldwide, should be a health research priority. A genetic approach may indeed be useful, but large collaborative studies will also be needed.

## Supporting Information

Figure S1 Funnel Plot of Studies of *ACE-I/D* Polymorphism and Preeclampsia ORs for outcome using a per-allele model. Studies in bold are those published in non-English-language journals.
(38 KB PPT)

Figure S2 Cumulative Synthesis of Studies of *ACE-I/D* Polymorphism and Preeclampsia OR (random effect model) for outcome using a per-allele model.
(36 KB PPT)

Protocol S1 *ACE-I/D* Polymorphism and Preeclampsia Risk: Evidence of Small-Study Bias (68 KB DOC)

Alternative Language Abstract S1 Translation of the Abstract into Spanish Translation by N. C. Serrano, Universidad Autónoma de Bucaramanga, Bucaramanga, Colombia.
(25 KB DOC)

Alternative Language Abstract S2 Translation of the Abstract into Chinese Translation by D. Wang, Medical Statistics Unit, London School of Hygiene and Tropical Medicine, London, United Kingdom.
(33 KB DOC)

### Accession Numbers

The SNP database (http://www.ncbi.nlm.nih.gov/SNP/) single-nucleotide polymorphism discussed in this paper is an insertion/deletion variant (rs1799752) referenced to the *ACE* gene with the GenBank (http://www.ncbi.nlm.nih.gov/Genbank/) accession number J04144.

## Abbreviations

ACE: angiotensin-converting enzyme
*ACE-I/D*: angiotensin- converting enzyme gene
CI: confidence interval
OR: odds ratio
