# Supplementary material for: Angiotensin-Converting Enzyme I/D Polymorphism and Preeclampsia Risk: Evidence of Small-Study Bias
Source: PLoS Med. 2006 Dec 26;3(12):e520. doi: 10.1371/journal.pmed.0030520 (PMC1716194; doi:10.1371/journal.pmed.0030520)
Supplement: Supplementary file 1 [file pmed.0030520.sd001.doc]

**Angiotensin-Converting Enzyme Insertion/Deletion Polymorphism and Preeclampsia risk: evidence of small-study bias.**

**Authors:** Norma C. Serrano MD, MSc.^1^; Luis A. Díaz, MD, MSc.^1^; Maria C. Páez, MD.^1^; Clara M. Mesa, MD,MSc.^2^; Rodrigo Cifuentes, MD, PhD.^3^; Alvaro Monterrosa, MD.^4^; Adriana González, MD.^5^; Liam Smeeth, MRCGP, MSc, PhD.^6^; Aroon D. Hingorani, FRCP, PhD.^7^; Juan P. Casas, MD.^6,7^

**Appendix**

**Methods section**

***Genotyping***

PCR Conditions: Reactions were performed with 12,5 pmoles of each primer: sense oligo 5´ CTG GAG ACC ACT CCC ATC CTT TCT 3´and anti-sense oligo: 5´GAT GTG GCC ATC ACA TTC GTC AGA T 3´ (Gibco BRL) in a final reaction volume of 25 µL, containing 3mM MgCl_2_, 400 µM of each dNTP , 50 mM KCl, 10 mM Tris-HCl (ph 8.5) and 1 unit of *Taq* DNA Polymerase (PROMEGA Corporation). The DNA was amplified for 30 cycles with denaturation at 94 ^0^C for 1 min, annealing at 65 ^0^C for 1 min, and extension at 72 ^0^C for 1 min using a PTC-100 thermal cycler (MJ Research). PCR products were electrophoresed in 2% agarose gel and analyzed by ethidium bromide stain under ultraviolet light. Genotype interpretation was based on the length of the PCR products: 190 and 490 base pairs for the deletion (D) and insertion (I) alleles, respectively.

To avoid mistyping of ID subjects as DD due to insufficient amplification of I allele, all samples genotyped as DD by the standard amplification procedure were then subjected to a second independent PCR amplification with a primer pair that recognizes an insertion-specific sequence (5'-TGG GAC CAC AGC GCC CGC CAC TAC-3'; 5'-TCG CCA GCC CTC CCA TGC CCA TAA-3' [Gibco BRL]), with identical PCR conditions except for an annealing temperature of 67 ^0^C.

**Appendix. Table I.** Genotype and allelic frequency in all ethnic group, by ethnic background, and by site of recruitment.

|  | **Genotype** | | | *HWE*  *χ^2^( p)* | ***Allelic frequency*** | |
| --- | --- | --- | --- | --- | --- | --- |
|  | *DD* | *ID* | *II* |  | *D* | *I* |
| **Ethnic background**  White-Hispanics  Cases (n = 63)  Controls (n = 141)  Afro-Caribbeans  Cases (n = 117)  Controls (n = 176)  Amerindians  Cases (n = 9)  Controls (n = 11)  Mestizas (Mixed)  Cases (n = 476)  Controls (n = 718) | 12 (19.0%)  33 (23.4%)  21 (17.9%)  39 (22.2%)  3 (33.3%)  5 (45.5%)  104 (21.8%)  153 (21.3%) | 37 (58.7%)  84 (59.6%)  75 (64.1%)  116 (65.9%)  4 (44.4%)  6 (54.5%)  266 (55.9%)  401 (55.8%) | 14 (22.2%)  24 (17.0%)  21 (17.9%)  21 (11.9%)  2 (22.2%)  0  106 (22.3%)  164 (22.8%) | 5.34 (0.0197)  19.41 (<0.0001)  19.41 (<0.0001)  10.36 (0.0013) | 61 (48.4%)  150 (53.2%)  117 (50.0%)  194 (55.1%)  10 (55.6%)  16 (72.7%)  474 (49.8%)  707 (49.2%) | 65 (51.6%)  132 (46.8%)  117 (50.0%)  158 (44.9%)  8 (44.4%)  6 (27.3%)  478 (50.2%)  729 (50.8%) |
| **Recruitment place**  Bogotá  Cases (n = 53)  Controls (n = 87)  Bucaramanga  Cases (n = 153)  Controls (n = 363)  Cali  Cases (n = 29)  Controls (n = 11)  Cartagena  Cases (n = 331)  Controls (n = 408)  Medellín  Cases (n = 99)  Controls (n = 177) | 8 (15.1%)  21 (24.1%)  28 (18.3%)  62 (17.1%)  6 (20.7%)  1 (9.2%)  73 (22.1%)  104 (25.5%)  25 (25.3%)  42 (23.7%) | 28 (52.8%)  42 (48.3%)  81 (32.0%)  195 (53.7%)  14 (48.3%)  4 (36.4%)  196 (59.2%)  249 (61.0%)  63 (63.6%)  117 (66.1%) | 17 (32.1%)  24 (27.6%)  44 (28.8%)  106 (29.2%)  9 (31.0%)  6 (54.5%)  62 (18.7%)  55 (13.5%)  11 (11.1%)  18 (10.2%) | 0.097 (0.7559)  3.108 (0.0824)  0.075 (0.7849)  24.03 (<0.0001)  21.29 (<0.0001) | 44 (41.5%)  84 (48.3%)  137 (44.8%)  319 (43.9%)  26 (44.8%)  6 (27.3%)  342 (51.7%)  457 (56.0%)  113 (57.1%)  201 (56.8%) | 62 (58.5%)  90 (51.7%)  169 (55.2%)  407 (56.1%)  32 (55.2%)  16 (72.7%)  320 (48.3%)  359 (44.0%)  85 (42.5%)  153 (43.2%) |

*χ^2^* value for departure from Hardy-Weinberg equilibrium.

**Appendix. Table II.** Estimate of the effect for ACE-I/D polymorphism and preeclampsia according to recruitment centre participating in the study according to the presence or not of evidence for departure from Hardy-Weinberg equilibrium. The model used for this sub-group was a per-allele model, the main model of comparison.

| **Groups evaluated** | **Unadjusted OR**  **(95% CI)** | **Model-1 OR**  **(95% CI)** | **Model-2 OR**  **(95% CI)** |
| --- | --- | --- | --- |
| Centres in Hardy-Weinberg equilibrium  (Bogota, Bucaramanga, Cali) | 0.98  (0.78, 1.24) | 0.98  (0.77, 1.26) | 0.85  (0.69, 1.06) |
| Centres in Hardy-Weinberg diseqiulibrium  (Medellin, Cartagena) | 0.84  (0.68, 1.03) | 0.85  (0.69, 1.05) | 0.96  (0.74, 1.24) |

**Appendix. Figure 1.** Funnel plot of studies of ACE-I/D polymorphism and preeclampsia. Odds ratios for outcome using a per-allele model. Studies in bold are those published in non-English-language journals.

**Appendix. Figure 2.** Cumulative synthesis of studies of ACE-I/D polymorphism and preeclampsia. Odds ratios (random effect model) for outcome using a per-allele model.
