# Supplementary material for: Angiotensin-Converting Enzyme I/D Polymorphism and Preeclampsia Risk: Evidence of Small-Study Bias
Source: PLoS Med. 2006 Dec 26;3(12):e520. doi: 10.1371/journal.pmed.0030520 (PMC1716194; doi:10.1371/journal.pmed.0030520)
Supplement: Supplementary file 2 [file pmed.0030520.sd002.doc]

**Polimorfismo Insersión/Delección de la Enzima Convertidora de Angiotensina y riesgo de Preeclampsia: evidencia de sesgo de estudios pequeños**

# Resumen

**Introducción:** La activación inadecuada del sistema renina-angiotensina, puede hacer parte del desarrollo de preeclampsia. El polimorfismo inserción/delección en el gen que codifica para la enzima convertidora de angiotensina I (I/D-ECA), ha mostrado estar consistentemente asociado con diferencias en la actividad de la ECA. Sin embargo, estudios previos de asociación entre la variante I/D-ECA y preeclampsia han tenido un bajo poder individual para detectar un riesgo real conferido por el genotipo.

**Métodos:** Se realizó un estudio de casos y controles en 1711 mujeres embarazadas jóvenes no relacionadas (665 con preeclampsia, 1046 controles gestantes normotensas), captadas en 5 ciudades de Colombia. Se obtuvo sangre materna para genotipificar el polimorfismo I/D-ECA. Para evaluar la fuerza de la asociación entre la variante I/D-ECA y el riesgo de preeclampsia, se utilizó un modelo de regresión logística para obtener la razon relativa (Odds Ratio: OR) crudo y ajustado con su intervalo de confianza del 95% (IC95%). Se realizó un meta-análisis de todos los estudios publicados hasta Febrero de 2006, que evaluaran la variante I/D-ECA en preeclampsia.

**Resultados**: El modelo aditivo (por alelo D) mostró un asociación nula entre la variante I/D-ECA y el riesgo de preeclampsia (OR crudo= 0.95 [IC95%: 0.81,1.10]) en el nuevo estudio de casos y controles. Resultados similares fueron obtenidos después de ajustar por variables de confusión (OR ajustado por alelo = 0.90 [IC95%: 0.77,1.06]) y usando otros modelos genéticos de herencia. El meta-análisis (2596 casos y 3828 controles a partir de 22 estudios) mostró un OR por alelo de 1.26 (1.07,1.49). Un análisis estratificado por el tamaño del estudio mostró una disminución de los OR hacia el valor nulo en la medida que el tamaño de la muestra de los estudios aumenta.

**Conclusión**: El pequeño incremento nominal observado en el riesgo de preeclampsia asociado con el alelo D del gen de la ECA, es debido probablemente a sesgo de pequeños estudios, similar a lo observado en enfermedad cardiovascular. Una evaluacion rigurosa de los orígenes de la preeclampsia usando una aproximación genética requiere probablemente el establecimiento de un consorcio de colaboracion para obtener tamaños de muestra adecuados.

**Author of translation:** Norma C. Serrano, MD, MSc. Universidad Autonoma de Bucaramanga, Colombia.
