# Supplementary material for: Angiotensin-Converting Enzyme I/D Polymorphism and Preeclampsia Risk: Evidence of Small-Study Bias
Source: PLoS Med. 2006 Dec 26;3(12):e520. doi: 10.1371/journal.pmed.0030520 (PMC1716194; doi:10.1371/journal.pmed.0030520)
Supplement: Supplementary file 3 [file pmed.0030520.sd003.doc]

**血管紧张素转换酶的插入/缺失多态型和惊厥症危险性:**

**小样本研究具有偏差**

**摘	要**

**研究背景:** 肾上腺素-血管紧张素系统的活性异常可导致惊厥症的发生。已有研究表明,血管紧张素转换酶 (ACE) I的插入/缺失多态型和ACE的活性相关，然而已有关于ACE不同基因型和惊厥症的研究都是独立进行的，从而得出某基因型的发病危险性结论, 是似是而非的。

**方法:** 对1711名哥伦比亚城市孕妇(665名患惊厥症，1046名健康并作为对照) 进行病例对照研究，采集母体血液用作ACE-I的基因定型，用logistic回归模型，以概率 (OR) 和 95%CI为指标，评价不同基因型和惊厥症发生危险性之间的相关性，并用荟萃分析对2006年2月前发表的所有有关基因型和惊厥症发生危险性研究的数据进行分析。

**结果:** per-*D*-allele模型显示, ACE-I基因型和惊厥症危险性之间无相关性(crude OR= 0.95 [95%CI: 0.81, 1.10])，当调整独立等位基因(per-allele)模型的 OR= 0.90 [0.77, 1.06]，以及用其它的一些遗传学模型也得出类似结果。对22份研究报告中包括2596个病例，3828个对照的数据进行荟萃分析，表明 per-allele OR为 1.26 (1.07, 1.49)。对研究样本大小的分析表明, 随样本的增大，OR减小趋于零相关。

**结论:** 已有研究观察到的所谓惊厥症和ACE等位基因之间的相关性很可能是由于研究样本小导致的偏差，和心血管疾病研究的观察结论类似。用某个遗传学方法可靠评价惊厥症的病因，需要通过团体的合作建立一个足够大的样本。

**关键词:** 惊厥症, 血管紧张素转换酶, 基因, 多态型, 荟萃分析, 病例对照研究, 哥伦比亚

# Author of translation: Duolao Wang. BSc, MSc, PhD. Medical Statistics Unit, London School of Hygiene and Tropical Medicine.
